# Using Indoor Movement Complexity in Smart Homes to Detect Frailty in Older Adults: Multiple-Methods Case Series Study

**DOI:** 10.2196/77322

**Published:** 2026-01-02

**Authors:** Katherine Wuestney, Diane Cook, Catherine Van Son, Roschelle Fritz

**Affiliations:** 1 College of Nursing, Washington State University, Pullman, WA, United States; 2 School of Electrical Engineering and Computer Science, Washington State University, Pullman, WA, United States; 3 Betty Irene Moore School of Nursing, UC Davis Health System, 2570 48th St, Sacramento, CA, 95817, United States, 1 916 734 2145

**Keywords:** entropy, complexity, smart home, movement, frailty

## Abstract

**Background:**

The theory of complexity in aging indicates that the complexity of sensor-derived physiological and behavioral signals reflects an older adult’s adaptive capacity and, in turn, their frailty. Smart homes with ambient sensors offer a unique opportunity to longitudinally explore the complexity of older adults’ indoor movement in a real-world setting. Here, we introduce a computational method to estimate behavior complexity from sensor data. We further conduct a multiple-methods case series to explore the relationship between entropy-measured smart home data complexity and older adult frailty.

**Objective:**

This study aims to explore the relationship between entropy-measured ambient sensor data complexity and frailty in independent community-dwelling older adults.

**Methods:**

The nature of older adults’ indoor movement complexity is measured by quantifying the entropy of smart home data. Overall, 11 cases with persons aged 65 years and older were drawn from an ongoing smart home study to illustrate the method. We assessed weekly frailty for these cases using the Clinical Frailty Scale. For corresponding time ranges, we measured the complexity of smart home data using a fixed-width sliding window and an entropy-based complexity index (Rényi Complexity Index) built on a Universal Sequence Map (USM-Rényi). Descriptive statistics and graphical analysis were used to describe intraindividual frailty and sensor complexity change.

**Results:**

The complexity of sensor-observed indoor movement does change over time in older adults as quantified by the computational method. In some individuals, these changes track with health transitions and frailty progression. The trends and monotonicity of complexity trajectories varied between cases. Overall, 3 of the cases demonstrated a negative association between frailty and complexity, while the association was not as clear for the other cases.

**Conclusions:**

The complexity of older adults’ smart home data is highly diverse. Changes in health and frailty influence indoor movement complexity. Although the findings suggest a relationship between frailty and complexity, confounding factors, such as home layout, visitors, external events, and technology disruptions, may influence sensor signals.

## Introduction

### Background

Frailty is a critical public health challenge among older adults globally. It is characterized as a clinically identifiable state of diminished physiologic reserve and heightened vulnerability to stressors and affects. An estimated 10%‐15% of community-dwelling individuals aged 65 years and older experience frailty, with the prevalence escalating to 51% among those aged 90 years and older [1]. This multifactorial syndrome, encompassing multiple impairments such as physical weakness, exhaustion, slow gait, low activity levels, and unintentional weight loss, elevates risks for adverse outcomes like falls, hospitalizations, functional decline, and mortality [1,2]. When unaddressed, frailty imposes substantial economic burdens, with frail older adult women incurring as much as 184% the health care cost of nonfrail older adult women [3,4]. Poor outcomes related to frailty strain health care systems and diminish quality of life [3-5].

Most older adults prefer to remain in their own homes and communities as they age. Despite this desire, age-related frailty and its sequelae remain a threat to their independence and quality of life. Older adults who are frail are more likely to present with atypical, nonspecific symptoms of acute illness, which include immobility, instability, incontinence, weakness, and delirium [6]. This can put them at risk for poorer outcomes if such atypical signs are treated as the primary problem rather than merely the manifestation of underlying, seemingly unrelated illnesses. Thus, while frailty is a significant issue, it also functions as a gateway to a wide array of other salient health issues for older adults [7].

Two urgent challenges face older adults who wish to age in place: (1) needing validated methods to detect incipient frailty at home and (2) determining the best way to analyze these data for predicting frailty that focuses on the efforts of early intervention strategies [8]. This study addresses both challenges by exploring complexity as a feature of frailty in smart home sensor data. In the context of aging, the term “complexity” is often used to describe difficult problems that must be mitigated, making care more daunting [9]. While complex behavior is often seen as a challenge in caregiving, from a systems theory perspective, too little complexity may indicate diminished physiological adaptability. The theory of complexity in aging asserts that complexity is a direct indicator of the health of physiologic systems and aging reduces this complexity, resulting in frailty [10].

Sensors are ubiquitous in our world, and this reality is accompanied by an increasing interest in discovering indicators of human health using these sensors. These indicators serve a similar function as conventional biological and imaging biomarkers with less reliance on expensive lab equipment, visits to remote sites for time-consuming tests, or physically invasive procedures [11]. Digital biomarkers are valuable components of geriatric telehealth and precision medicine since these technologies support continuous, longitudinal, remotely delivered measurement of intraindividual changes in older adults’ health [12]. Among this class of markers are behavior markers created from continuously collected sensor data, which open substantial opportunities to explore the complex dynamics of aging in an ecologically valid, real-world setting.

In this study, we enlist digital biomarkers to explore the relationships between behavior and frailty. This is increasingly important because the number of persons aged 80 years and older is expected to triple between 2020 and 2050 [13]. With the rise in age-related frailty and incidence of chronic conditions, meeting the health needs of older adults is increasingly burdensome. A review of unobtrusive frailty digital biomarkers concluded that passive infrared motion sensors, especially as part of a smart home, are the most promising type of embedded ambient sensor for detecting frailty [14]. Smart homes were promoted for their potential to uniquely inform individual responses to disease or treatment.

Older adults prefer digital biomarker technologies that minimally impose on their lifestyles [15]. From this perspective, digital behavior markers derived from completely passive monitoring (eg, ambient sensors embedded in residential environments) offer advantages over those measured via semipassive or active monitoring (eg, wearable sensors that must be routinely charged and positioned) [14]. Smart homes represent a passive biomarker technology that consists of ambient sensors to monitor movement and door interactions, combined with a computing infrastructure to collect, organize, and store the data [16]. The resulting time series data can be analyzed to understand the smart home resident’s health status.

### Prior Work

The theory of complexity in aging hypothesizes that measuring the complexity of a person’s sensor-derived signals can indicate the underlying state of an older adult’s adaptive capacity [17,18]. We analyze a person’s behavioral signal complexity as an indicator of their adaptive capacity or functional reserve, referring to the capacity of their physiological and behavioral systems to maintain or regain function when perturbed. In a complex-systems view of aging, this reserve depends on the multiscale dynamics that are present between the system components [19]. These dynamics support homeostasis, the process that maintains internal stability while adapting to change.

In earlier work, researchers have investigated the use of multiscale entropy (MSE) to quantify complexity across time scales for physiological data. Bizovska et al [20] used MSE and Shannon entropy to analyze gait complexity as a mechanism for predicting fall risk in older adults. Castiglia et al [21] investigated the selection of MSE parameters that yield the best predictive probability in differentiating subjects with Parkinson disease from healthy subjects based on trunk acceleration patterns. Gao et al [22] use distribution entropy, which calculates the complexity of signal pattern distribution within a phase space representation, to determine whether pulse rate complexity is associated with corresponding cognitive decline in older adults.

Frailty is hypothesized to be an emergent state that arises from a critically dysregulated complex system [9]. In other words, the system dynamics may erode with aging and disease, causing complexity to decline and frailty vulnerability to increase. Evidence from cross-sectional studies suggests this process can be observed as a change in complexity in a diverse range of physiological and behavioral signals. For example, lower blood pressure interbeat interval complexity, when the beat-to-beat pattern becomes simpler and more uniform, is associated with greater frailty and dementia risk [23]. Similarly, lower moment-to-moment center-of-pressure complexity, such as simpler, more regular sway, during balance tasks is associated with increased future incidence of falls [24]. Reduced complexity of spontaneous brain activity, measured via the blood oxygenation signal, is associated with slower gait speed [25], and lower physical activity complexity and variance are associated with greater self-reported frailty [26] and mortality risk [27]. These examples suggest that reduced signal complexity may be a generalizable marker of physiologic decline.

Prior studies have measured the complexity of smart home data [28,29]. These earlier studies included complexity as one of a set of variables input to machine-learning models that were trained for specific tasks such as detecting visitors or predicting in-home movement [30,31]. Little is known regarding how within-person complexity, in isolation from other variables, evolves in relation to health outcomes over the long term. In a study by Schutz et al [28], Shannon entropy of refrigerator use was one of the strongest predictors of frailty (*r*=−0.25). However, this analysis did not explore the evolution of complexity over time. A study by Takahashi et al [32] examined activities over a 2-year period and found that increased activity diversity manifested an inverse relationship with frailty. The findings support our hypothesis, but they are based on survey data rather than analysis of passively observed activity patterns.

Two prior studies applied complexity measures to data collected by the Center for Advanced Studies in Adaptive Systems (CASAS), the same data collection infrastructure used in this study. Specifically, Wang et al [30] estimated complexity using compression-based estimators to establish a theoretical limit on the predictability of indoor human mobility. In an earlier study by Gopalratnam and Cook [33], CASAS smart home data were analyzed with a Lempel-Ziv compression-based incremental parser to predict the resident’s next interaction with the home. Although the smart home sites and analysis goals differed from this study, the prior work established the use of such behavior analyses from smart home data.

The common approach to predicting frailty leverages sources such as electronic health records and manually collected clinical data [34]. However, wearable sensors are increasingly accessible and offer a mechanism for passively sensing and detecting frailty [8]. Many frailty studies that analyze wearable data focus on predicting physical frailty components such as slowness and inactivity. These studies extract gait parameters such as cadence and indicators of time spent walking and standing [35,36]. One study instead analyzed Fitbit data that were collected while individuals performed an upper extremity function test [37]. While the primary component of these analyses is accelerometry, Merchant et al [38] combine these parameters with heart rate to analyze scripted movements such as sit-to-stand, walk, and climb stairs.

Wearable sensors have demonstrated the ability to sense and quantify changes in movement parameters that are associated with frailty. We focus here on monitoring activity and detecting frailty using ambient sensors in smart homes. Ambient sensors impose no user burden. Sensors collect data for multiple years on a charge, which results in continuous, uninterrupted monitoring of in-home behavior as a person’s health status changes. Using wearable sensors, consistent multiday wear is challenging, and adherence varies with demographics and cognition [39]. While wearable sensors provide direct access to heart rate and gait parameters, the smart home sensors contribute context-rich information about location traces, sleep and wake routines, and activity patterns that are not easily modeled from wearable data [40]. Because we want to monitor uninterrupted longitudinal behavior patterns, we focus this analysis on data collected in smart home settings.

To address this knowledge gap, we present an exploratory case series investigating how the complexity of older adults’ indoor movement patterns, as captured by the CASAS smart home, changes over time in relation to changes in their health status. Considering that this relationship between complexity and frailty has been observed across a diverse range of seemingly unrelated physiologic and behavioral signals, we hypothesize that changes in the complexity of time series obtained from smart home sensors are similarly associated with changes in health status and frailty of the older adult smart home occupant.

The case series design prioritizes investigation of intraindividual interpretation and allows us to integrate each participant’s clinical narrative into the analysis. To promote replication of methods and application to new data, we make the analysis and visualization tools publicly available for the community to use in the calculation of sensor-derived behavior complexity.

## Methods

### Overview

We performed a multiple-methods exploratory case series, combining participant narrative and qualitative nursing data with complexity analysis of smart home sensor time series data to contextualize intraindividual changes in complexity of indoor movement. A case series analysis was chosen because the method is useful for exploring intricate, real-world issues in novel ways, especially when triangulating data from different sources to discover differences and similarities across similar cases. The method fosters a more nuanced, valid, and actionable understanding of the cases under study.

### Participants

We used secondary data from sensors installed in the homes of community-dwelling older adults between October 2016 and December 2022 as part of the ongoing clinician-in-the-loop smart home research study [41]. To be included in the clinician-in-the-loop study, participants had to be aged 60 years and older, have at least 1 chronic condition, and had to be proficient in English. For this case study series, we applied the additional criteria of living alone without pets for the entire duration of the data collection and collected a minimum of 9 months of smart home data. Cases were further excluded if a majority of the days and sensors were missing. The resulting sample consisted of 11 cases, representing a balance between stable participants and those who exhibited frailty transitions. For this case series, each participant is considered as 1 case. Among these participants, cases 8 through 10 exhibited constant frailty scores, while the others experienced frailty that fluctuated throughout the data collection.

Participant cases included in the present analysis lived in independent living apartments in continuing care retirement communities. Most cases’ ages were in the range of 80 to 89 years, although 2 were aged 70‐79 years and 1 was aged 90‐99 years. All included cases identified as non-Hispanic White, and 7 of the cases identified as women. Information summarizing participants, their chronic health conditions, and their home characteristics is provided in Table 1.

**Table 1.** Demographics and data characteristics for each participant case.

Case	Age^a^ (years)	Sex	Home type	Sensors	Days	Window size^b^	Chronic conditions
1	80‐89	Female	1-bedroom apartment	15	310	25,159	CV^c^, NM^d^, Pain^e^
2	70‐79	Female	1-bedroom apartment	13	416	63,419	CV, Pulm^f^, Pain
3	80‐89	Female	3-bedroom duplex	20	366	19,714	CV, Pulm, NM
4	80‐89	Female	1-bedroom apartment	13	349	33,358	CV, Pulm, NM
5	80‐89	Male	1-bedroom apartment	15	629	60,800	NM, Pain
6	80‐89	Female	1-bedroom apartment	12	571	28,951	CV, Pulm, NM, Pain
7	80‐89	Female	1-bedroom apartment	14	385	28,182	Pain, CI^g^
8	70‐79	Male	2-bedroom duplex	22	330	49,303	CV, NM, Pain
9	80‐89	Female	Studio apartment	11	354	35,310	CV, Pain, CI
10	90‐99	Male	1-bedroom apartment	12	264	18,951	CV, NM, Pain
11	80‐89	Male	1-bedroom apartment	13	286	34,903	CV, Pain

a To preserve privacy, age is given as a range.

b Sliding window size was determined by the maximum biweekly count of sensor messages (excluding OFF and CLOSE) observed in the participant’s data.

c CV: cardiovascular.

d NM: neuromuscular.

e Pain: chronic pain.

f Pulm: pulmonary.

g CI: cognitive impairment.

### CASAS Smart Home

**Figure 1.**
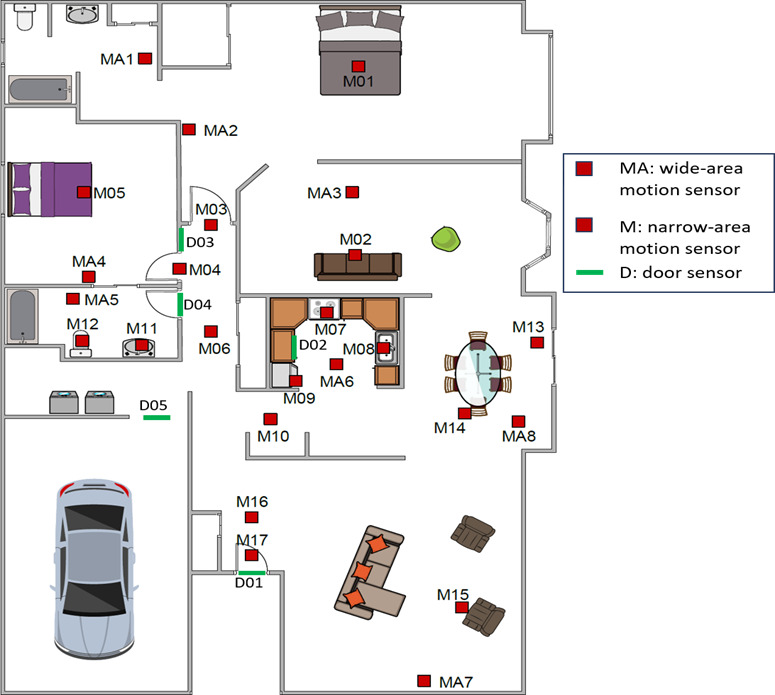
Location of sensors in a Center for Advanced Studies in Adaptive Systems smart home.

The CASAS smart home contains passive infrared motion detectors, light, magnetic door use, and temperature sensors. These sensors were installed strategically throughout each house to capture activity in critical locations (Figure 1). At least 1 motion detector with a 360^o^ view was installed in each room. Additional motion detectors with a narrower field of view (approximately 1 m in diameter) were positioned in areas of high use, such as the bed, sinks, toilet, and frequented furniture (eg, preferred living room chair). Because floor plans, furniture layouts, and daily routines differed across each home, the number of sensors installed for included cases ranged from 11 to 22.

CASAS sensors send messages containing their readings to a middleware layer resident on a Raspberry Pi [16]. Architecture components communicate using a Zigbee wireless mesh. The middleware publish and subscribe manager allows hardware components to publish and receive messages. And annotates sensor readings with the corresponding sensor identifier and timestamp. All collected data are encrypted and securely transmitted to a password-protected server for storage and analysis.

We examined data collected from the passive infrared and door-use sensors. Each sensor samples the environment at 1.25 Hz. Rather than report the state at a constant frequency, the sensors record data when a change in state is sensed (eg, a door is opened, motion is detected). Once triggered, the sensor sends a message reflecting the new state to a central relay, which labels each message with the sensor identifier and timestamp, then transmits the data to a secured database. The resulting dataset is a timestamped series of binary messages (“ON” or “OFF” for motion sensors, “OPEN” or “CLOSE” for door sensors) indicating the time and location of the sensor reading in the home. Because an ON message from a motion sensor is followed by an OFF message (marking the end of movement within the sensor’s field of view or lack of activity for 1.25 s), both ON and OFF messages artificially inflated the regularity of the data sequence. Following previous literature measuring the entropy of smart home data [30,42], we excluded all OFF messages from motion sensors and all CLOSE messages from door sensors. Example deidentified CASAS datasets are available online [43].

### Clinical Data

For each participant, nurse researchers conducted an initial comprehensive geriatric assessment, including functional status in activities of daily living (ADL) and instrumental activities of daily living (IADL), current health diagnoses, health history, medications, fall history, psychosocial supports and family presence, assistive device use, review of body systems, and personal demographic history. Participants then received weekly follow-up telehealth calls from a nurse researcher to assess for any changes in health or function from baseline. Weekly nursing data included, but was not limited to, vital signs, pain, sleep quality, psychosocial well-being (including the presence of visitors), changes in ADL and IADL status, and a brief review of physiologic systems and daily routines [41].

Although frailty was not measured as part of the primary data collection, the clinical data collected during the study provided information to retrospectively estimate weekly frailty using the Clinical Frailty Scale (CFS) [44]. The CFS is a 9-point scale (1=very fit to 9=terminal illness) designed to guide a clinician in assessing a holistic picture of a person’s frailty status using elements of a comprehensive geriatric assessment, including overall activity level, functional dependence, and management and control of chronic condition symptoms [45]. Two CFS-trained nurse scientists reviewed the clinical data for each participant and assigned a frailty score for each week of data collection (Table 2). Changes in ADL and IADL independence, use of a new assistive device, and descriptions of increasing fatigue or “slowing down” were the most common health changes associated with an upward shift in the participant’s CFS score.

**Table 2.** Example Clinical Frailty Scale codebook with scores for case 4.

Week^a^	Date (2017)	CFS^b^ score	Rationale
45	July 24	5	No change
46	July 31	5	No change
47	August 7	5	Decreased activity, increased weeping lower legs
48	August 14	6	“I have to be careful not to fall”
49	August 21	6	Considering assisted living but hiring in-home help
50	August 28	6	No change
51	September 4	6	Losing weight, legs improving
52	September 11	6	Legs continue weeping due to heart failure
53	September 18	6	Began using pursed-lip breathing, moving less
54	September 25	6	Doctor’s visit, medication change
55	October 2	6	Legs improving, taking diuretic

a Weeks 1‐44 (CFS score: mean 4.9, SD 0.33; range 4‐5); weeks 56‐60 (CFS score: mean 6.4, SD 0.89; range 6‐8).

b CFS: Clinical Frailty Scale.

### Data Preprocessing

Because smart home data were collected in real-world settings over extended periods, we needed to address missing and noisy data. We screened each participant’s sensor data for evidence of sensor malfunctioning, extended absences, and other issues. Periods associated with participant absence for more than one night (eg, vacation or hospitalization) were excluded from the analysis. Additionally, any periods where all sensors did not report readings, regardless of explanation, were excluded. Periods with no messages from a given sensor were cross-referenced with battery data from that sensor to confirm whether the absence was due to a change in behavior or a sensor malfunction. Sensors missing >50% of the observation time over one or more consecutive days were excluded. Sensors missing more than 50% of the observation period were either excluded or they were included, and the time associated with that sensor’s absence was excluded.

The varying size of the homes and the corresponding number and density of sensors impact the scale of Rényi Complexity Index (RényiCI) values we observe in each home. A cross-sectional study would require that sensors be grouped into larger, consistently sized sets or that the values be normalized. For this study, we are interested in within-home RényiCI changes, so no adjustments are made to the per-home RényiCI scales. Because the sensors report binary state (motion ON or OFF, door OPEN or CLOSED), the raw sensor values are not normalized.

Some of the participants included in this study were enrolled during the onset of the COVID-19 pandemic, which had a dramatic global impact on daily activities. For those participants, if the majority of a participant’s data were collected after the pandemic onset, data from before March 16, 2020, were excluded. Similarly, for participants with most data collected before the pandemic, we excluded data from March 16, 2020, onward.

Data cleaning included the removal of sensor data from analysis for sensors sending “error” signals, which can occur when low battery health or technical issues occur during installation. Only 2 homes were affected by this: case 1 (dining room area sensor, hallway, bathroom sink, and door for the primary bedroom) and case 5 (entry door, refrigerator, and bathroom area sensor). After data cleaning procedures were applied, the series of timestamped sensor messages was coded based on the sensor identifiers, resulting in a time series of discrete (categorical) sensor states. These discrete-valued series were then used to compute the complexity of sensor state transitions over time.

### Complexity Measurement

Understanding patterns in human behavior, especially those that signal changes in health or frailty, requires tools that quantify how predictable or irregular those behaviors are over time. One such method is based on entropy, a way of measuring complexity or unpredictability in a sequence of events. Almeida and Vinga [46] introduced a technique to calculate this complexity using a Universal Sequence Map (USM). This approach turns a sequence of events (eg, daily activities recorded in a smart home) into a set of coordinates in a multidimensional space. These coordinates reflect how often and in what sequence specific symbols (eg, messages from home sensors) occur relative to one another. Once the sequence has been mapped to this space, the method estimates how densely packed these points are in space using the kernel density estimation statistical technique. The resulting density provides insight into whether the behavior is highly repetitive (low complexity) or highly varied (high complexity). Highly repetitive behavior may, for example, reflect a person moving primarily between the living room and bathroom each day. A more complex behavior will vary the daily sequence and perhaps more frequently introduce additional areas, including the guest room, the garage, and the front door to leave the home.

**Figure 2.**
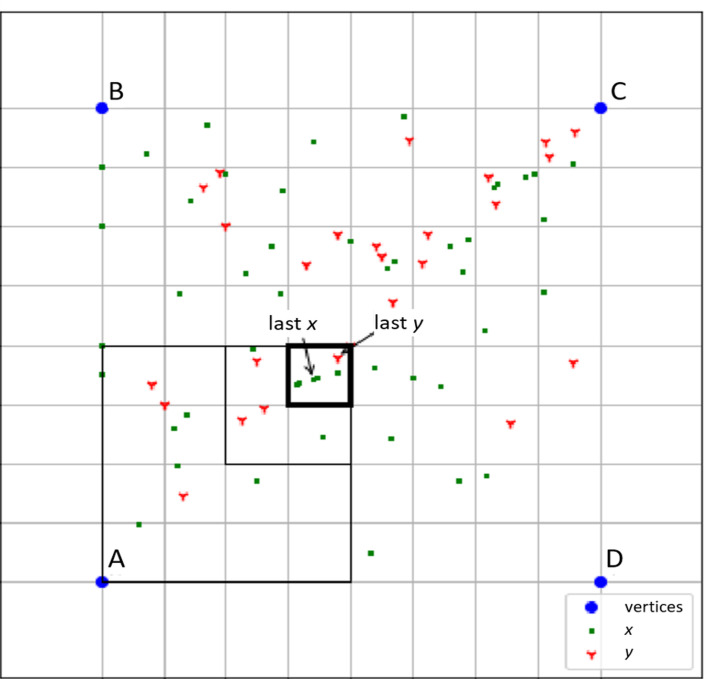
Plot of 2 sequences in a Universal Sequence Map. The last 4 symbols of sequence x are *ACCA,* and the last 4 of sequence y are *CCCA*. The highlighted subquadrant contains the coordinate of the sequences’ last symbol, *A*.

Figure 2 illustrates the process of creating a USM. Unique sensor readings are converted to symbols (A-D). USM coordinates are calculated by assigning each unit symbol in the sequence to a position in a multidimensional space. The positions are defined so that each symbol is equally distant from the others, ensuring that no symbol is biased in how the space is structured. The number of dimensions of the space is chosen so that each distinct symbol can be uniquely represented using binary digits. The sequence is processed forward (considering prior context) and backward (considering subsequent context), and the 2 resulting maps are merged to capture bidirectional structure in the behavior.

The resulting space creates a unified framework to measure the complexity of sequences from the resulting coordinates. To quantify the complexity of these mapped sequences, Vinga and Almeida [47] introduced a method that computes Rényi entropy, a generalization of Shannon entropy, from the density of the USM coordinate distribution. This approach is particularly effective for relatively short sequences, such as those representing daily behavior in smart homes.

Since the idea of entropy was introduced in information theory, many variations have been introduced to measure complexity in different contexts. These measures vary by the type and quantity of data they process, their sensitivity to noise, and their assumptions about the underlying state space distribution. An ideal measure of sensor-based time series complexity is one that tends toward a minimum value for both deterministic and random sequences while handling varying alphabet sizes and being sensitive to changes in complexity over short sample lengths. Rényi entropy of USMs was selected for our analysis as a method that meets these constraints.

A key strength of this method is its flexibility: it can emphasize either common or rare patterns, depending on how the parameters are configured. Importantly, the frequency of any subsequence of any length can be estimated by analyzing how dense different regions of the USM space are. The kernel size (ie, the size of the region considered) controls the length of the subsequences being emphasized. We use this principle to estimate Rényi entropy at multiple scales, where each scale corresponds to a different behavioral timespan or sequence length. This flexibility enables a multiscale view of behavioral complexity, which we refer to as the RényiCI. An in-depth tutorial and code are provided online [48].

### Statistical Analysis

Because this case series investigates how the complexity of motion sensor transitions, representing indoor movement trajectories, evolves over time, we computed RényiCI for each participant using a sliding window approach with a fixed window size, *n*. The actual RényiCI values will shift with the number of sensors in the space and the window size; thus, the values should be examined for change within a single home across multiple time points. Higher RényiCI values indicate more complex behavior, while lower values suggest simpler, more predictable patterns. The sliding window method evaluates the time series in overlapping segments: starting with the first *n* data points, it computes summary statistics, shifts the window forward by a set number of steps, and repeats the process.

To ensure each window captured both routine cyclic behaviors (eg, weekly housekeeping) and short-term variations, we defined each participant’s window size as the maximum number of sensor messages observed within any 2-week period (Table 3). The window was advanced using a step size equal to one-quarter of the window size.

**Table 3.** Sliding window statistics for each case. Runs test results were omitted as all resulted in *P* values <.001.

Sliding window			RényiCI statistics					ρ^a^	*P* value
Case	Count	Days, median (IQR; max)	Mean (SD)	CoV^b^^c^	Median (IQR)	KS^d^	*P* value		
1	70	16.6 (15.5 to 17.2; 18.9)	−45.95 (0.26)	.006	−45.89 (−46.07 to −45.79)	0.10	.41	0.11	.48
2	66	24.7 (21.7 to 26.1; 30.8)	−38.85 (0.57)	.015	−38.65 (−39.09 to −38.47)	0.21	<.001	0.29	.06
3	80	16.3 (15.4 to 16.9; 18)	−61.97 (0.23)	.004	−61.94 (−62.18 to −61.78)	0.11	.29	0.12	.36
4	73	18.1 (16.8 to 19.2; 21)	−38.91 (0.27)	.007	−38.93 (−39.13 to −38.74)	0.09	.60	0.01	.96
5	121	18.1 (17.4 to 18.8; 30.7)	−44.66 (0.19)	.004	−44.68 (−44.81 to −44.51)	0.09	.23	−0.68	<.001
6	121	16 (15.5 to 16.6; 18.4)	−36.32 (0.10)	.003	−36.3 (−36.4 to −36.25)	0.09	.34	0.26	<.001
7	82	16.8 (15.6 to 17.7; 20.5)	−42.74 (0.14)	.003	−42.76 (−42.84 to −42.66)	0.07	.77	0.42	<.001
8	52	17.1 (16.1 to 18.1; 20.1)	−70.13 (0.20)	.003	−70.18 (−70.26 to −70.01)	0.12	.38	—^e^	—
9	73	18.3 (17.7 to 19; 20.6)	−33.07 (0.13)	.004	−33.07 (−33.15 to −32.99)	0.05	.98	—	—
10	49	18.9 (18 to 19.5; 22.6)	−36.64 (0.25)	.007	−36.64 (−36.84 to −36.43)	0.12	.47	—	—
11	65	15.5 (15 to 16.2; 18)	−39.58 (0.24)	.006	−39.58 (−39.78 to −39.35)	0.10	.53	0.24	.09

a Spearman rank correlation.

b CoV: coefficient of variance.

c Coefficient of variance was computed as the SD/mean.

d KS: Kolmogorov-Smirnov distance.

e Correlation is not provided because CFS is a constant.

To examine how the complexity of patterns relates to frailty status, we visualized RényiCI values using time series plots and categorical scatter (jitter) plots. Because RényiCI values can vary in scale depending on the number of sensors and the window size, we applied normalization within each case to enable comparison. To assess temporal fluctuations in complexity, we also computed the first-order difference of the normalized RényiCI sequence: $∆RenyiC{I`}_{t}={RenyiCI`}_{t}-RenyiC{I`}_{\left\{t-1\right\}}$. Here, $∆RenyiC{I`}_{t}$ represents the change in normalized complexity between consecutive windows.

To evaluate whether these complexity estimates varied systematically over time (in comparison to random changes in complexity), we applied Kolmogorov-Smirnov (KS) tests and runs tests to each participant’s sequence of RényiCI values under the null hypothesis of randomness. The KS test checks whether the complexity values follow a normal distribution, as would be expected with random data. The runs test looks at the order of values in the sequence, rather than just the distribution, to determine if they appear in nonrandom patterns. Computation of USM-based RényiCI values was conducted in Python (version 3.9; Python Software Foundation) using our pyusm library [48]. This open-source package is publicly available and includes tools for computing USM, USM-Rényi, and generating 2D USM visualizations.

Finally, to resolve ambiguous quantitative results, sequential explanatory techniques were used. Quantitative results were reviewed alongside frailty scores assigned to each week of nursing narrative documentation, which included written text about participants’ physical and functional health recorded during weekly phone calls and monthly home visits. RényiCI complexity values were compared to recorded CFS scores. Lower complexity values combined with higher CFS scores meant the participant was frailer.

### Ethical Considerations

The Washington State University Institutional Review Board approved the presented secondary analysis (protocol 18764) and parent study (protocol 15412). All participants provided informed consent, and their data were deidentified and securely managed for analysis. Participation was voluntary and without compensation.

## Results

### Distributional Characteristics and Statistical Testing

**Figure 3.**
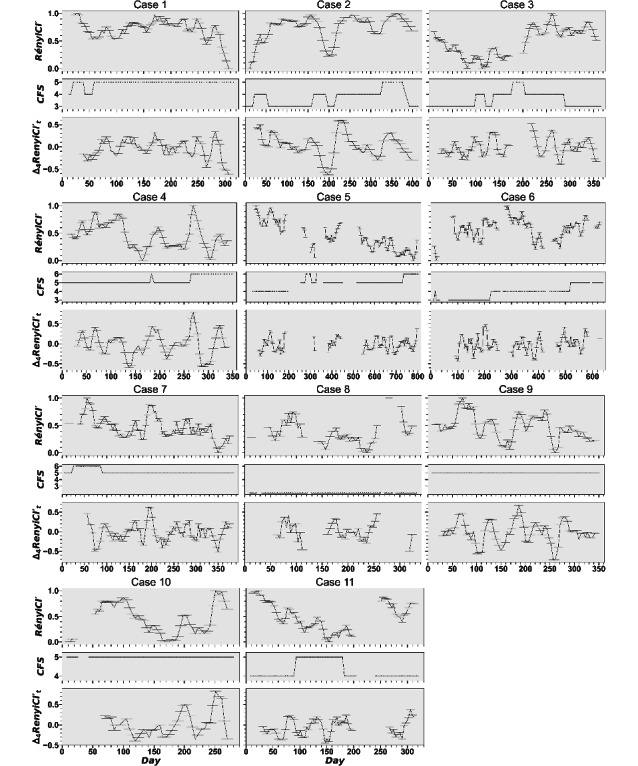
Plots of complexity, frailty, and complexity change as a function of time. Lower values reflect less complexity. Δ_4_RényiCI' represents the value difference between sliding windows at times *t* and *t-4*. CFS: Clinical Frailty Scale; RényiCI: Rényi Complexity Index.

**Figure 4.**
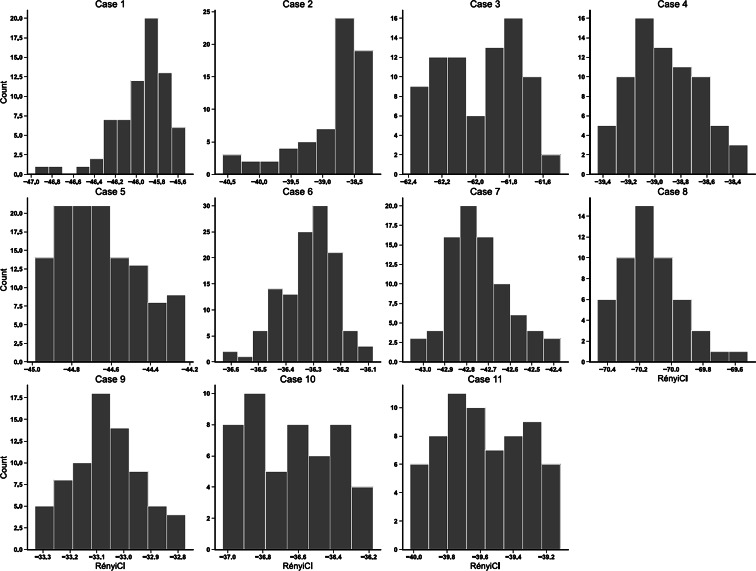
Histograms of RényiCI values.

Figure 3 presents time series plots of normalized RényiCI, frailty scores, and first-order differences in normalized RényiCI for each case. In the plots, time is measured in observation days. Summary statistics of overall RényiCI, KS, and Spearman rank correlation values are reported in Table 3. The shape of the RényiCI distributions varied notably across cases. Case 9 exhibited the only unimodal, symmetric distribution (Figure 4), while the remaining cases showed skewness or kurtosis. Cases 1 and 2 were strongly left-skewed, while cases 3, 5, 10, and 11 displayed low kurtosis. Cases 3 and 11 also showed bimodal distributions. Despite this heterogeneity, only case 2 showed a statistically significant deviation from a random normal distribution (*P*=.006) based on the KS test of normality.

Overall, case participants ranged from “fit” (CFS=2) to “living with moderate frailty” (CFS=6), although the trajectories of frailty within each participant varied considerably (Figure 3). For example, cases 2, 3, 7, and 11 all experienced periods of elevated frailty but all recovered and returned to baseline by the end of observation. Cases 4, 5, and 6 were the only cases that increased in frailty over time, ending frailer than their baseline. Most cases exhibited 2‐3 transitions in frailty over time, with the extreme being case 2 with 7 frailty transitions.

While the goal of this study is not to directly infer CFS from RényiCI values, we note that Table 3 shows a significant correlation for all cases that have variable frailty scores. While they are significant, the correlations are mostly quite small. The overall correlation for all combined values is ρ=−.055 (*P*<.001). These results indicate that while a relationship between behavioral complexity and frailty can be observed, other factors must be considered when assessing a person’s frailty from smart home sensor readings.

As Figure 3 demonstrates, trajectories of sensor complexity were similarly varied. RényiCI values for cases 1, 5, 7, and 9 exhibited downward trends over time, while cases 3 and 6 demonstrated an overall positive trend. Case 11 showed a concave shape with a general downward trend in sensor RényiCI for the first half of the data, followed by a general upward trend. The remaining cases exhibited nonmonotonic fluctuations. In each case, RényiCI values and frailty trajectories aligned with frailty scores assigned by the CFS-trained researchers during qualitative processing of clinical data.

### Frailty-Complexity Associations

**Figure 5.**
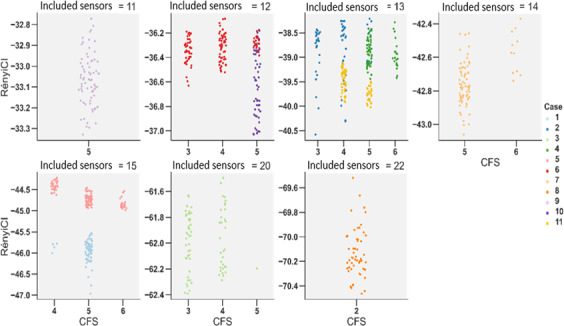
Categorical scatter (jitter) plot of RényiCI values by frailty, grouped by sensor count and case. CFS: Clinical Frailty Scale.

The relationship between frailty level and sensor data complexity also varied from case to case. Figure 5 shows jitter plots of RényiCI values by CFS score grouped by the number of sensors installed in the home. Cases 5 and 11 exhibited a negative trend between complexity and frailty, while cases 3 and 7 demonstrated a mostly positive trend. The range and mean of USM-Rényi values shift farther from 0 as the number of sensors in the home increases. The range of RényiCI for the home with the fewest sensors (11 sensors) spanned approximately −34 to −32, while in the home with the most sensors (22 sensors), the range extended from about −71 to −69.

Initial runs tests applied to the full sequence of RényiCI values were statistically significant (*P*<.001) for all participants, suggesting nonrandom temporal ordering. To reduce potential autocorrelation introduced by overlapping windows, we repeated the runs tests on a downsampled sequence using every fourth window. Under this condition, only cases 3 (*P*<.001) and 5 (*P*=.002) remained statistically significant.

To account for these differences, Table 3 also includes the coefficient of variation (CoV) that normalizes the RényiCI SD by the mean for each case. Cases 6, 7, and 8 exhibited the least amount of relative variability in RényiCI (CoV=0.003), while case 2 exhibited the highest (CoV=0.015).

The cases with statistically significant nonoverlapping runs tests, cases 3 and 5, also represented the clearest long-term monotonic trends. Case 3 demonstrated an overall increase in complexity between the start and end of her data, while case 5 demonstrated an overall decreasing trend. The only RényiCI distribution with a statistically significant KS test, case 2, had some of the most extreme variation among the cases, with an extremely left-skewed distribution and a coefficient of variation 5 times greater than the smallest coefficient of variation among the cases. To explore possible explanations for the diverse patterns of frailty and complexity trajectories observed, we compared the frailty and complexity trajectories of the cases with contextual information derived from the nursing assessment records.

Data selection for cases 2 and 3 started at the beginning of the COVID-19 pandemic shutdowns. The horizontal bars in Figure 3 represent sliding window durations (in days). For both cases, the shortest windows are at the beginning of the time series. The shorter sliding window durations during this period likely reflect increased in-home activity, with participants generating more sensor events due to spending more time indoors during COVID-19 lockdowns. However, where RényiCI increased steadily over the coming months for case 2, RényiCI decreased steadily for case 3 (Figure 3).

Cases 2 and 3 also showed pronounced shifts in RényiCI midway through the observation period. For both cases, this period roughly correlates to a time period of hazardous air quality caused by continued wildfire smoke over the course of about a month. However, the steep dip in RényiCI for case 2 occurring between days 186 and 212 is short, while for case 3, the sharp increase in RényiCI around day 231 appears to be a vertical shift in their average complexity that continues for the rest of her data.

### Case Narratives

#### Case 2

A woman in her 70s with congestive heart failure and mild asthma. She was independent at baseline (CFS=3) and in stable health, which is consistent with the increasing RényiCI values plotted in Figure 3 at the beginning of data collection. This participant experienced 3 episodes of worsening fatigue and shortness of breath (days 20‐54, 160‐215, 258‐397), which impacted her ADLs and IADLs and contributed to transient increases in frailty. During the first episode, the nurse’s report indicated that “walking has been much more taxing on her this past week. She will walk around the building today but runs out of energy very quickly... her fatigue level has increased significantly over the past week.” During the second episode, the nurse reported “the last 3 days she noticed … more soa [sic, shortness of air] and tired[ness].” During the third episode, the nurse recorded a direct quote from the participant indicating that she had “absolute fatigue beyond anything I’ve experienced.” Two of these periods coincided with substantial troughs in RényiCI, suggesting alignment between behavioral simplification and functional decline. The primary movement patterns manifested in the CFS score and measured by RényiCI (ie, mechanisms of interest) were less in-home overall activity and less time spent out of the home on walks and social activities (frequency and duration). This case also had the highest variability in complexity and was the only one with a statistically significant KS test result. Possible gerontological clinical actions informed by these results include recommendations to follow up with the cardiologist, referral to a pulmonologist, and referral to senior services to determine whether the patient qualifies for in-home care support services.

#### Case 3

A woman in her 80s with cardiovascular disease and allergy-induced asthma. Initially independent, she experienced progressive health decline, including 2 hospitalizations for acute hypertension and dyspnea. Her frailty peaked after the second hospitalization during a period of wildfire smoke, when she relied full-time on a walker for ambulation. Nursing records include statements during this timeframe like “no energy, has not left the house since Thursday [4 d]” and “overall health is declining.” Following physical therapy, she recovered and reported no activity limitations by day 295. The nursing record indicated that she “went shopping” and had “several visitors over” across multiple days of the week. Her RényiCI trajectory reflected this pattern. As shown in Figure 3, the RényiCI values initially show a steady decrease aligned with the health issues. After she received treatment and improved her ambulation and functional independence, the RényiCI values showed a steady rise in complexity. Notably, this case showed a strong monotonic increase in RényiCI and passed the runs test even under downsampling. This result provides evidence that the pattern of increasing frailty, followed by improvement after treatment, is distinct and nonrandom. The mechanism of interest impacting her RényiCI trajectory was a renewed increase in time spent out of the home (frequency and duration) concurrent with an increase in the number of visitors. The case exemplifies how RényiCI trajectories could help clinical gerontologists understand treatment efficacy through novel remote patient monitoring tools that include sensor monitoring and associated behavior patterns.

#### Case 5

A man in his 80s with Parkinson disease. He began with mild frailty (CFS=4) and was independent but slowed by symptoms. Over time, he required increasing assistance with ADLs and IADLs. Nurses recorded that he began to require assistance “getting compression sock on in the morning and off at night” and “needing help with laundry and housekeeping” and that his daughter began assisting with bill paying. He experienced multiple hospitalizations and rehabilitation stays and ultimately progressed to moderate frailty (CFS=6). The moderate frailty score was based on the nurse reporting “unsteady gait” and that he “has cracked ribs from a fall last week” and his “symptoms of PD [are] increasing, [and] noticeable upon observation.” His RényiCI trajectory followed a corresponding decline, with complexity peaking early and then falling across successive rehabilitation episodes. This case also exhibited a significant runs test and a clear downward trend in complexity. The mechanism of interest in this case was more overall time spent in his recliner chair, more nighttime sleeping in the recliner chair, and the decreased time spent out of the home (frequency and duration). This case illustrates how RényiCI trajectories may support automated smart home monitoring aimed at detecting increasing frailty upstream so interventions can be implemented.

#### Cases 4 and 10

Both cases involved sustained or increased caregiving over time. In case 4, RényiCI peaked just before caregiving began and declined thereafter. Case 10, who had consistent caregiving throughout, showed generally lower complexity than case 6, who lived alone with the same number of sensors. These comparisons suggest that increased caregiving frequency does not necessarily lead to increased behavioral complexity as measured by RényiCI. Older adults with consistent professional caregiving are likely to experience slower rates of decline due to the intentionality of caregiving, which aims to extend independence through building physical, functional, and cognitive strength. Findings could inform care planning and resource allocation.

## Discussion

### Principal Results

This study introduces and applies a novel entropy-based algorithm, the RényiCI, to quantify behavioral complexity from smart home sensor data in older adults. Using a USM framework with multiscale Rényi entropy, our method captures subtle temporal dynamics in sensor-derived movement sequences. In this exploratory case series, within-person indoor-movement complexity, as exhibited by RényiCI values, fluctuated over time. In several cases, these fluctuations coincided with frailty changes.

Across 11 participants, we observed diverse complexity trajectories, ranging from steady increases, steady declines, and concave patterns to nonmonotonic fluctuations. Case-level analysis revealed that greater fluctuations in complexity were frequently aligned with periods of functional decline or recovery. Notably, 2 cases (3 and 5) exhibited statistically significant nonrandom patterns in complexity over time, confirmed by runs tests on downsampled data, and showed clear monotonic trends in behavior complexity that matched health trajectories. Only one case (2) showed a RényiCI distribution that deviated significantly from normality, corresponding with extreme within-person variability and periods of worsening frailty. Scatter plots further revealed heterogeneous associations between complexity and frailty, with both positive and negative trends across cases. Importantly, increased caregiver presence was not associated with greater behavioral complexity, suggesting that RényiCI may reflect intrinsic changes in individual functional capacity rather than external support.

Changes in CFS scores fluctuated in alignment with changes in RényiCI values for some cases, like 2, 3, 6, and 7. These cases may suggest that changes in frailty do impact the person’s behavioral routine and regularity. However, in cases 8 through 10, we observed changes in RényiCI values despite the lack of change in frailty scores. This observation highlights the fact that our findings provide 1 set of indicators of changes in frailty, but should not be analyzed in isolation. Other factors, such as visitors, seasonal effects, and external events, can also impact behavioral routines. These should be controlled for when examining frailty as a function of changes in RényiCI.

While observed repetitive behavior may correlate with frailty, the relationship is not one-to-one. Reduced complexity of movement often, but not always, aligns with frailty progression. Repetitive behavior can signal frailty because it reflects narrowed activity routines, reduced introduction of new routine elements, and corresponding reduced adaptability. At the same time, we note that complexity is multifactorial. Other influences, such as visitors in the home, home layout, and external events, also affected the entropy measures. The results showed nonmonotonic relationships in those cases. To interpret complexity, it is therefore best to consider an individual over time rather than compare cross-sectionally. Moreover, interventions aimed at slowing the impact of frailty on maintaining independence, like a smart home that projects RényiCI trajectories, would be more helpful for older adults living alone. Mechanisms of interest become difficult to automatically recognize in multiresident homes where ambient sensors detect movement from all residents.

### Limitations

Several factors impacted the interpretation and generalizability of our findings. First, entropy-based measures like RényiCI are inherently sensitive to sample length and the number of sensors deployed in a participant’s home. To prioritize intraindividual validity, we customized the sliding window size for each participant using a fixed number of sensor messages (*n*), rather than a fixed time duration. This approach allowed for consistent comparisons within individuals but introduced variability in the time span covered by each window, both within and across cases, limiting our ability to analyze complexity as a direct function of chronological time. Future work could develop correction factors for RényiCI to account for sample length, enabling the detection of periodic, seasonal, or event-driven patterns in indoor behavior.

Relatedly, interindividual comparisons were constrained by differences in sensor configurations across homes. Participants varied in the number and placement of sensors, affecting both the density of event data and the scale of RényiCI values. Standardizing sensor deployments in future studies would facilitate more robust cross-participant comparisons and support investigation into whether home-level sensor complexity systematically relates to frailty markers at the population level.

The impact of the COVID-19 pandemic further complicates interpretation. Several participants were enrolled during or shortly after the onset of pandemic-related lockdowns, which led to changes in daily routines, increased time spent indoors, and potentially long-term shifts in behavior and social support. These behavioral changes may have altered both the complexity of movement and its relationship to frailty. Additionally, one period of the study coincided with prolonged hazardous air quality due to regional wildfires, which may have further restricted participants’ movement and contributed to abrupt changes in sensor complexity. Such exogenous events likely altered daily routines independent of health. We therefore interpret Rényi changes within homes and in the presence of annotated event periods. We also provide event-excluded sensitivities to reduce confounding.

Sensor noise and dropout also presented challenges. While preprocessing steps excluded known periods of sensor failure or participant absence, subtle forms of sensor drift or inconsistent message delivery could still introduce noise into the RényiCI estimates. Further improvements to sensor reliability and the integration of sensor health metrics into complexity analysis pipelines would strengthen future research.

In terms of statistical methods, the runs test was useful in identifying nonrandom patterns in behavioral complexity over time, but it is not well-suited to detecting more complex temporal structures such as oscillatory or nonlinear trends. Future research may benefit from time series models drawn from signal processing or machine learning that can more precisely characterize evolving behavioral dynamics.

Frailty measurement also posed a limitation. Because frailty was not a primary outcome in the parent study, we relied on retrospective CFS scoring based on weekly nursing reports. This limits temporal precision and may miss subtle fluctuations in functional status. Larger-scale studies using prospectively collected frailty data, including both clinician-reported and self-reported measures, could reveal more detailed associations between complexity and health.

Finally, this sample was racially and culturally homogeneous, limiting the generalizability of our findings. RényiCI analyses should be interpreted as a within-home monitoring signal rather than a cross-sectional diagnostic tool. As efforts to diversify smart home research populations expand, it will be essential to explore whether the relationships between sensor-derived behavioral complexity and frailty differ across racial, cultural, and socioeconomic groups. Inclusive, representative samples are critical to ensuring that digital biomarkers are both effective and equitable.

### Conclusions

Detection of incipient frailty in community-dwelling older adults is a key component to supporting their independence. The findings in this study demonstrate that RényiCI, as a passive and unobtrusive complexity metric, offers a promising tool for monitoring functional health changes in aging populations and may help enable early detection of frailty in real-world settings. The PyUSM software package developed for this analysis is publicly available and supports future application of this method in diverse behavioral monitoring contexts. These findings support the potential of entropy-based digital behavior markers to unobtrusively monitor intraindividual health changes and capture early signs of frailty in aging-in-place.

Future enhancements of this analysis may reveal additional factors that influence change in indoor movement complexity and inform how the complexity of smart home data may inform clinical practice. For example, significant departures of RényiCI values from a person’s complexity baseline may trigger a nurse call or follow-up when integrated into a remote monitoring or telemonitoring system. In routine care, weekly summaries of the analysis would support triage and help care providers select appropriate actions. Additionally, when performing a functional assessment of an individual, a summary of the complexity trend augments traditional frailty analysis to improve assessment and treatment options. Future work will also emphasize analytical validity (repeatability and robustness across sensors and windowing), clinical validity (prospective prediction of frailty transitions), and clinical use (impact on downstream outcomes such as unplanned care, falls, and functional decline) for diverse homes and populations.

Additionally, future work should focus on integrating RényiCI in machine learning predictive modeling as a high-level feature to assist with identifying meaningful digital biomarkers [49]. Other temporal activities associated with frailty (eg, walking speed, ADL, and IADL behaviors) could also be integrated to optimize frailty classifications. Machine learning integration of features from RényiCI values that signal possible increasing frailty will support nurses and caregivers in providing timely interventions, thereby potentially extending independence and optimizing older adults’ outcomes.

## Abbreviations

ADL: activity of daily living
CASAS: Center for Advanced Studies in Adaptive Systems
CFS: Clinical Frailty Scale
CoV: coefficient of variance
IADL: instrumental activity of daily living
KS: Kolmogorov-Smirnov
MSE: multiscale entropy
RényiCI: Rényi Complexity Index
USM: Universal Sequence Map
